# Using genetic tools to estimate the prevalence of non‐native red deer (*Cervus elaphus*) in a Western European population

**DOI:** 10.1002/ece3.3282

**Published:** 2017-08-18

**Authors:** Alain C. Frantz, Frank E. Zachos, Sabine Bertouille, Marie‐Christine Eloy, Marc Colyn, Marie‐Christine Flamand

**Affiliations:** ^1^ Musée National d'Histoire Naturelle Luxembourg Luxembourg; ^2^ Fondation faune‐flore Luxembourg Luxembourg; ^3^ Natural History Museum Vienna Vienna Austria; ^4^ Département de l'Etude du Milieu naturel et agricole Service Public de Wallonie Gembloux Belgium; ^5^ Institut des Sciences de la Vie Université catholique de Louvain Louvain‐la‐Neuve Belgium; ^6^ CNRS‐UMR 6553 Université de Rennes 1 Paimpont France

**Keywords:** anthropogenic impact, microsatellites, non‐autochthonous animals, wildlife forensics, wildlife management

## Abstract

Game species like the red deer have been subjected to anthropogenic impacts for centuries. Translocations are often carried out—sometimes illegally—not only for sporting purposes, but also to increase trophy quality, reduce inbreeding, or mitigate bottlenecks after excessive persecution. Apart from the blurring of large‐scale genetic structure, translocations without adequate quarantine measure risk introducing pathogens into potentially immunologically naïve populations. It is therefore important to understand the frequency of clandestine translocations. Identification of non‐autochthonous animals and their potential origin is often difficult and, in red deer, has been hampered by the lack of large‐scale genotypic datasets for comparison. In the present study, we make use of a recently published European‐wide microsatellite dataset to detect and quantify the presence of non‐autochthonous red deer in a large population sample (*n* = 1,780) from Central Europe (Belgium). Using factorial correspondence analysis, assignment tests and Bayesian clustering algorithms we arrive at an estimate of 3.7% non‐autochthonous animals (or their descendants). Some of these animals were assigned to a nearby French population and may have immigrated into Belgium naturally, but the large majority must have been introduced by humans. Our analysis pointed to the British Isles and Germany/Poland as the potential origin of many introduced deer, regions known to have been source populations for translocations in Europe and beyond. We found evidence for recreational hunters using carcasses from farmed deer to fulfill mandatory hunting quotas. Our study is the first to quantify the extent of human‐mediated introductions in a European game species at such a large scale with large and representative sample sizes.

## 1 INTRODUCTION

The genetic structure of large mammal species, particularly those inhabiting areas with high human population density, is affected by natural and anthropogenic processes. The former include, among others, species‐specific dispersal behaviors and demographic changes in the wake of climate change, for example, during the Pleistocene when populations retreated to glacial refugia and subsequently expanded again (Hewitt, 2000). The latter comprise habitat fragmentation, persecution and, particularly in game species, selective hunting and translocations. Translocations, perhaps more than other factors, are able to blur natural geographic patterns of genetic diversity and relationships, and reviews on their frequency (Apollonio, Scandura, & Sprěm, 2014; Linnell & Zachos, 2011; Niethammer, 1963) suggest that anthropogenic impacts on genetic structuring are often as strong as natural processes.

Over past centuries, the red deer (*Cervus elaphus*) has arguably been one of the most important game species in Europe—and as a cervid with treasured antler trophies is known to have been impacted by humans for a long time (e.g., Hartl, Zachos, & Nadlinger, 2003). Studies on mitochondrial DNA sequences (Ludt, Schroeder, Rottmann, & Kuehn, 2004; Niedziałkowska et al., 2011; Skog et al., 2009) and microsatellites (Zachos et al., 2016) have shown the large‐scale genetic structure of European red deer to be shaped by the Late Pleistocene and Holocene glacial–interglacial cycles. Specifically, there are three genetic groups in Europe based on mtDNA: a western lineage from Iberia through France, the British Isles, Scandinavia, and Central Europe to Poland and Belarus (designated A); an eastern lineage in the Balkans and north to southern Central and eastern Europe (C); and a third lineage confined to the Tyrrhenian islands of Corsica and Sardinia and Northern Africa (B). The suture zone between the western and eastern lineages seems to run from Austria eastwards to Belarus and the Baltic States (Fickel et al., 2012; Krojerová‐Prokesová, Baranceková, & Koubek, 2015; Niedziałkowska et al., 2011). However, it remains unclear for many regions whether the occurrence of both lineages is natural or due to reintroduction of red deer after local extirpation. The large‐scale pattern of the three lineages that are geographically separated, however, strongly suggests that the natural genetic structure at the level of glacial refugial lineages is still present in red deer.

Apart from the species’ European‐wide phylogeography, local or regional red deer stocks have also been intensively studied from a population genetic point of view, often taking into account human impacts (Carranza, Salinas, de Andrés, & Pérez‐González, 2016; Frantz, Hamann, & Klein, 2008; Haanes, Røed, Flagstad, & Rosef, 2010; Haanes, Røed, Mysterud, Langvatn, & Rosef, 2010; Hoffmann, Johannesen, & Griebeler, 2016; Kuehn, Haller, Schroeder, & Rottmann, 2004; Kuehn, Schroeder, Pirchner, & Rottmann, 2003; Niedziałkowska, Jędrzejewska, Wójcik, & Goodman, 2012; Zachos, Althoff, Steynitz, Eckert, & Hartl, 2007). Some of these studies have identified clear phylogeographic outliers (e.g., a Sardinian haplotype in the British Isles, Nussey, Pemberton, Donald, & Kruuk, 2006; a few more phylogeographic outliers can be found in Skog et al., 2009), which is conclusive evidence of long‐distance translocations. Translocations of ungulates throughout Europe have been common for centuries (see Apollonio et al., 2014 and references therein). Apart from the blurring of natural structures, translocations also imply the risk of introducing pathogens into potentially immunologically naïve populations. Red deer can function as reservoirs for a variety of diseases, for example, bluetongue disease and bovine tuberculosis or foot‐and‐mouth disease (Ferroglio, Gortázar, & Vicente, 2011; Linden, 2012; Linden et al., 2010). It would therefore be important to understand the frequency of clandestine translocations.

To date, there are few studies directly aiming at identifying the genetic signature of translocation events. Probably, the most extensive such study was that of Frantz et al. (2006) who were able to prove the illegal introduction of a small number of deer into a local population in Luxembourg. In general, this kind of analysis is difficult as human‐mediated introductions cannot be unequivocally distinguished from natural immigration from a neighboring, but genetically differentiated population. Also, if the origin of a translocated animal is to be determined, DNA samples from ideally all possible source populations need to be available. Because until recently no European‐wide dataset based on high‐resolution markers such as microsatellites was available, genetic studies on translocations have long been hampered. In this study, we present an in‐depth analysis of regional red deer populations in Belgium—where since 1994 no private individual is legally allowed to translocate wildlife—based on 13 microsatellite loci and 1780 samples and compare this dataset to the recently published European‐wide dataset (Zachos et al., 2016) to identify non‐indigenous genotypes and to arrive at a quantitative estimate on how large the proportion of potentially translocated individuals is, thus testing claims that few if any European red deer populations are free from introductions (Hartl et al., 2003).

## 2 MATERIALS AND METHODS

### 2.1 Sample collection and laboratory work

**Figure 1:**
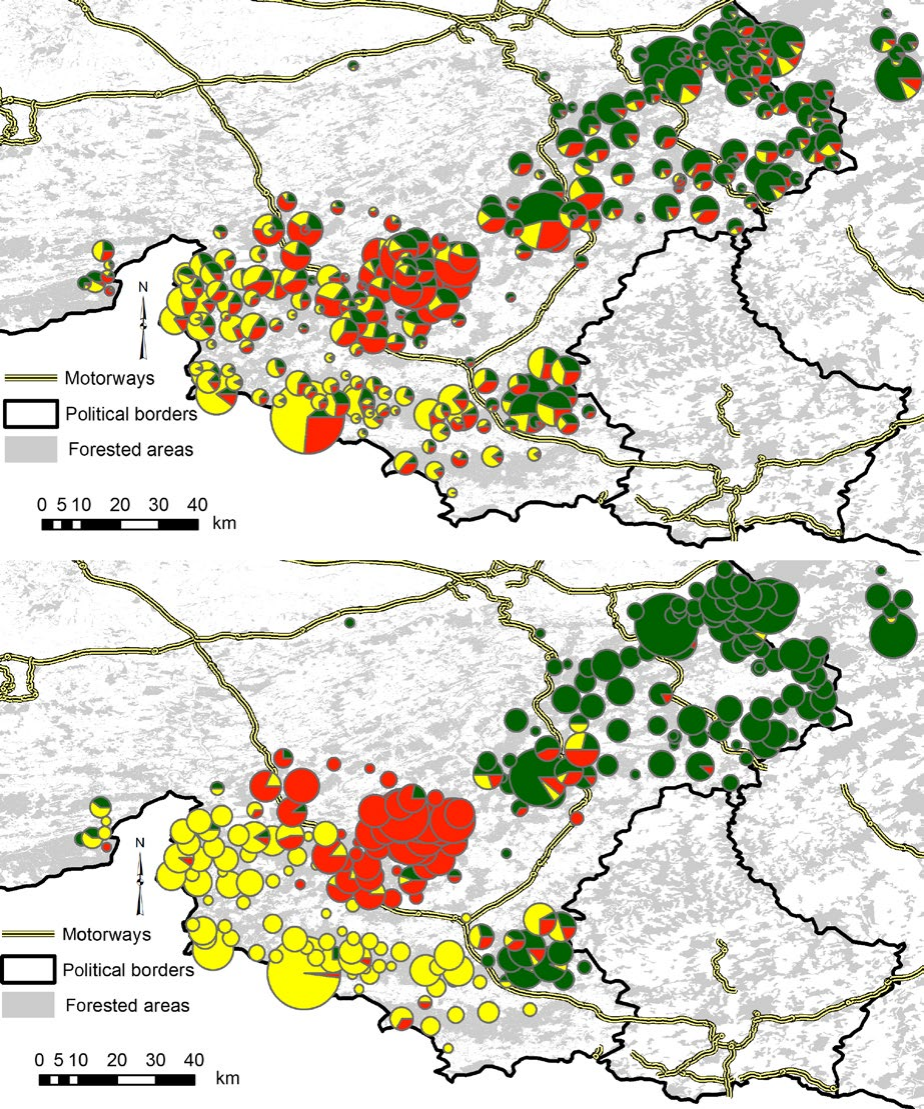
Geographic origin of Belgian red deer samples included in this study and location of the genetic subpopulations inferred using the STRUCTURE (top) and spatial BAPS (bottom) algorithms. The size of the pie charts indicates the number of samples collected from a locality, whereas the pattern of the pie chart indicates the identity of the genetic clusters

Between 2003 and 2009, we collected tissue samples from 1,733 red deer from the southern Walloon part of Belgium, as well as 47 samples from the neighboring Eifel region of Germany (Fig. 1; see also Frantz et al., 2012 who used about half of our present samples). For ease of reference, we will refer to the dataset as “Belgian” or “Walloon,” despite the few German animals. The total forested area of Wallonia amounts to approximately 5,000 km^2^, which corresponds to one‐third of the total area of the region. Human population density in the southern part of Wallonia, where our study was based, was mostly between 20 and 90 inhabitants⁄km^2^, depending on the municipality (Thomas, Frankhauser, & Biernacki, 2008). In 2014, the Walloon red deer population was estimated to have contained approximately 11,000 individuals (Direction de l'Etat Environnemental 2014). Samples were collected during legal hunts from harvested animals. The center of the forest management unit (a so‐called “triage”) where an individual was harvested was recorded as its sampling location.

DNA was extracted using a chloroform‐based extraction method (Doyle & Doyle, 1990). Samples were genotyped using 13 microsatellite loci (BM1818, Cer14, CSPS115, CSSM14, CSSM16, CSSM19, CSSM22, CSSM66, ETH222, Haut14, ILSTS06, INRA35, and MM12; for references see Kuehn et al., 2003) in three multiplex polymerase chain reactions (PCR) using the Qiagen Multiplex kit (Qiagen, Hilden, Germany). Detailed information on the PCR composition and reaction times can be found in Dellicour et al. (2011). We obtained a complete 13‐loci genetic profile for 1,746 of the 1,780 deer, with the remaining profiles having genotypes at a minimum of 11 loci. In all, 13 animals had a missing genotype at locus ILSTS06. Reactions were performed using a Verity thermocycler (Applied Biosystems, Warrington, UK). PCR products were separated using an ABI 3100 automated DNA sequencer (Applied Biosystems), and the data were analyzed using GeneMapper version 3.7 (Applied Biosystems).

### 2.2 European reference data

**Figure 2:**
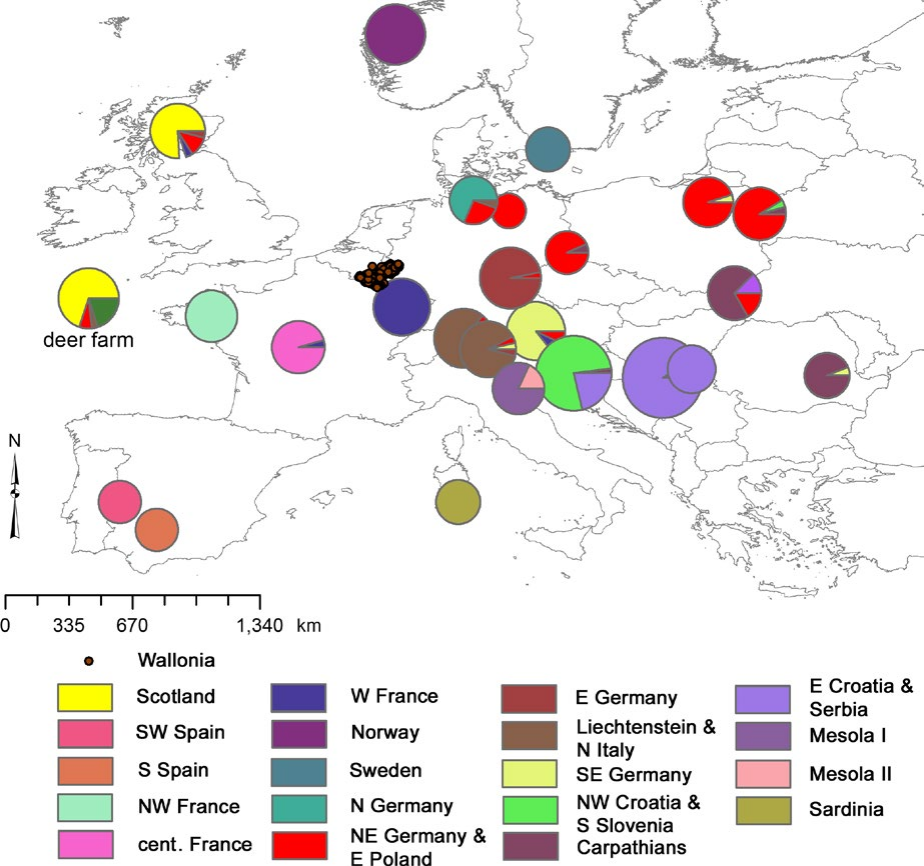
Geographic location of the European red deer reference populations and composition of the genetic populations inferred using the individual‐based BAPS algorithm from Zachos et al. (2016). The size of the pie charts indicates the number of samples collected from a locality, while the pattern of the pie chart indicates the identity of the genetic clusters. “Mesola II” was excluded from the analysis as it contained only six individuals. The entire deer farm (not indicative of geographic location) was considered to be a distinct reference population although its animals were assigned to different BAPS clusters. The locations of the Walloon red deer are indicated by individual sampling locations

We aimed to identify the source population of translocated deer, both to identify illegal introductions and animals that had dispersed naturally, using a European microsatellite reference dataset analyzed by Zachos et al. (2016). Not considering Belgian animals, it contained genetic profiles of 608 red deer from 26 locations throughout the continent (see Fig. 2), including 30 samples from a French deer farm (Boisgervilly). Both the Walloon and the European dataset were genotyped in the same laboratory using the same markers. We obtained a complete 13‐loci genetic profile for 571 of the 608 European deer, with the remaining profiles having genotypes at a minimum of 11 loci. Loci Cer14 and ILSTS06 did not amplify in 14 and 10 individuals, respectively. Using the individual‐based modal population mixture analysis implemented in BAPS v.5.4 (Corander, Waldmann, Marttinen, & Sillanpää, 2004), the European dataset was inferred to consist of 25 genetic populations (Zachos et al., 2016). Seven of these 25 clusters consisted of six individuals or less. These were excluded from the present analysis. Although the animals sampled at a deer farm were assigned to different genetic clusters, for the purposes of the present study, we created a separate reference population containing all the farmed deer. The European reference dataset therefore consisted of 596 non‐Belgian individuals belonging to 19 genetic clusters (18 BAPS‐defined partitions with *N *>* *6, plus the deer farm; Fig. 2).

### 2.3 Translocation analysis of Belgian red deer

While not having any a priori suspects, we needed to remove genetic outliers to avoid their interference in the analysis of the population genetic structure of Wallonia, as well as in the calculation of exclusion probabilities. We therefore used GENETIX v.4.05.2 (Belkhir, Borsa, Chikhi, Raufaste, & Bonhomme, 2004) to perform a factorial correspondence analysis (FCA) to visualize the genetic distance between all 1780 tested red deer. The coordinates of the two‐first principal components were entered into ARCVIEW 3.3 (ESRI Inc., Redlands, CA, USA) and we used the Animal Movement Analyst Extension (Hooge & Eichenlaub, 1997) to remove the outlying 5% (*N *=* *89) of all individuals using the harmonic mean method (Dixon & Chapman, 1980). The individuals that were outliers in the FCA analysis were excluded from the analysis of the population genetic structure of red deer in Belgium (it was later formally tested whether these deer were non‐autochthonous using an exclusion test; see below).

We analyzed the population genetic structure of the whole Belgian dataset (minus outliers) using two different (Bayesian) genetic clustering algorithms. First, we analyzed the data using the program STRUCTURE v. 2.3.1 (Pritchard, Stephens, & Donnelly, 2000). To estimate the number of subpopulations (*K*), 10 independent runs of *K *=* *1–10 were carried out with 10^6^ Markov chain Monte Carlo (MCMC) iterations after a burn‐in period of 10^5^ iterations, using the model with correlated allele frequencies and assuming admixture. ALPHA, the Dirichlet parameter for the degree of admixture, was allowed to vary between clusters. After deciding on the most probable number of sub‐populations based on the log‐likelihood values (and their convergence) associated with each *K*, we calculated each individual's percentage of membership (*q*) for each cluster, averaging *q* over 10 runs. Individuals were assigned to the STRUCTURE cluster for which they had the highest *q* value. Finally, we also analyzed the data using the spatially explicit genetic clustering method that is implemented in the program BAPS v.6.0 (Corander, Sirén, & Arjas, 2008). In addition to the genetic data, the algorithm considers the specific geographic coordinates of each individual and modally assigns each individual to its population of origin. We performed 10 runs for each *K *=* *2–10. We calculated the average assignment proportions for each sampling location and mapped them using ArcGIS 10.3 (ESRI Inc., Redlands, CA, USA).

For each STRUCTURE‐ and BAPS‐defined cluster, we tested for the significance of heterozygote deficiency or excess using the Markov chain method in GENEPOP v.4.0 (Raymond & Rousset, 1995), with 10,000 dememorization steps, 500 batches and 10,000 subsequent iterations. The false discovery rate technique was used to eliminate false assignment of significance by chance (Verhoeven, Simonsen, & McIntyre, 2005). We tested each STRUCTURE cluster for linkage disequilibria among loci using an exact test based on a Markov chain method as implemented in GENEPOP 3.4. Since the presence of immigrants can lead to linkage disequilibria among loci can (Paetkau, Slade, Burden, & Estoup, 2004), we did not exclude linked loci from further analysis.

Throughout this study, we used GENECLASS 2.0.g (Piry et al., 2004) to calculate the probability of an animal belonging to a genetic population (exclusion probability) based on the Monte Carlo method of Paetkau et al. (2004). We simulated 10,000 multi‐locus genotypes and set the threshold for individual exclusion to 0.01. In wildlife forensics, a more stringent threshold for excluding animals from a population—such as *p *<* *.001—is considered necessary (Manel, Berthier, & Luikart, 2002), but an exclusion threshold of *p *<* *.01 is normally used in ecological studies to identify genetic immigrants (e.g., Aspi, Roininen, Ruokonen, Kojola, & Vila, 2006; Clark, Brown, Stechert, & Zamudio, 2008; Proctor, McLellan, & Barclay, 2005). Individuals were assigned to their most likely source population (assignment test) using the partial Bayesian approach of Rannala and Mountain (1997) implemented in GENECLASS.

In order to avoid false inclusion of non‐autochthonous individuals in the Belgian reference dataset (not including the 89 outliers), we first calculated the exclusion probability of each Walloon deer for the three STRUCTURE‐defined reference populations using a leave‐one‐out approach where each individual in turn is excluded from the populations during computation. Furthermore, for each Belgian deer (that was not an outlier), we calculated the probability of belonging to each European reference population and assigned it to its most likely Belgian or European source population. The following Belgian animals were considered to be non‐autochthonous and excluded from the reference dataset for further analysis: (1) deer that could be excluded with *p *<* *.01 from all STRUCTURE‐defined Belgian reference clusters, (2) animals that were assigned with a (total) score of >0.85 to one (or two) European reference population(s). Furthermore, reference animals that were assigned with a score of <0.85 to a Belgian population and with a score of >0.15 to a European reference population were considered to have at least one non‐autochthonous recent ancestor. We choose the 0.85/0.15‐threshold as a compromise between correctly estimating the number of admixed individuals (efficiency) and identifying admixed individuals (accuracy). According to simulations by Vähä and Primmer (2006), the accuracy of detecting admixed individuals increases with increasing *q* value, but the total number of admixed individuals is over‐estimated. Given the number of loci used (*N *=* *13) and the degree of genetic differentiation between the reference populations (see Zachos et al., 2016), a 0.90/0.10‐threshold would be most likely to provide a correct estimate for the number of admixed individuals, whereas a 0.80/0.20‐threshold would allow identification of all admixed individuals (Vähä & Primmer, 2006).

Finally, considering the same criteria to assess non‐native status, we identified non‐autochthonous individuals among the 89 FCA‐outliers by first calculating the exclusion probability of each animal for every reference cluster (Belgium and Europe) and then assigning them to their most likely population of origin. The harvest locations of the non‐native deer were mapped using ArcGIS 10.3.

## 3 RESULTS

**Figure 3:**
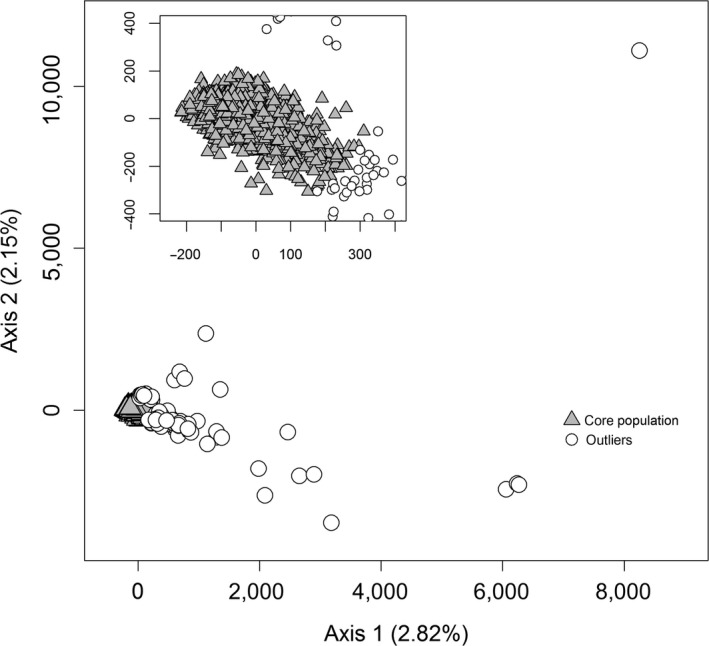
Factorial correspondence analysis of Belgian red deer (*N* = 1,780). The analysis was based on 13 microsatellite loci. The 89 (5%) outliers were identified using a harmonic mean method. The percentage of the total variation explained by each of the two axes is given. The inset magnifies the interface between the core population and the outliers

After excluding the 89 outliers identified using an FCA (Fig. 3), the log‐likelihood values generated by the STRUCTURE analysis supported the presence of three genetic clusters in the Belgian dataset (Fig. S1, Fig. 1). Similarly, the individual‐based spatial model implemented in BAPS inferred the presence of three geographically coherent genetic clusters in red deer from Wallonia (Fig. 1). Both programs roughly infer the same clustering solution. The spatial BAPS algorithm identified the deer sampled in the eastern half of the study area to form one genetic cluster. The deer in the western half formed two distinct genetic populations with the genetic discontinuities corresponding to a major motorway bisecting the study area (Fig. 1). The non‐spatial STRUCTURE algorithm suggested a more gradual differentiation between the three clusters.

While one to two loci deviated from Hardy–Weinberg equilibrium (HWE) in each STRUCTURE‐defined cluster after correcting for multiple tests, this was the case with zero to three loci in case of the BAPS‐derived clusters (Table S1). Locus CSSM66 deviated strongly from HWE in two of the three clusters, independently of the algorithm considered. It was therefore excluded from further analysis, which were based on the STRUCTURE clusters, since fewer loci overall deviated from HWE when considering the partition results of this algorithm (Table S1). After correcting for multiple tests, between one and three pairs of loci were in linkage disequilibrium in each STRUCTURE‐defined cluster.

Using the leave‐one‐out exclusion approach, eight deer from the Belgian reference dataset (89 outliers not included) could be excluded (*p *<* *.01) from all three STRUCTURE‐defined reference populations. Of these, seven could be excluded from all 19 European reference populations (Table 1; suggesting they originated from a non‐sampled population), while the eighth animal could be assigned to the NE German/E Poland reference cluster. A further five individuals could not be excluded from the Belgian reference clusters but were assigned to one (or two) European reference populations. Two of these five were assigned to the Eastern France population and could possibly have migrated naturally into the study area. Finally, 10 Belgian reference individuals were shown to be admixed with another European reference population (Table 1). Three of these 10 were admixed with Eastern France, the nearest European reference population. All these 23 (8 + 5+10) genotypes were excluded from the Belgian reference populations for further analysis.

**Table 1:** Identification of non‐autochthonous Belgian deer. We first removed 89 deer from the Belgian dataset that were outliers (5%) in an FCA analysis. For each animal, we then calculated the probabilities of it belonging to each of the three Belgian reference clusters dataset and to each of the 19 European reference populations by means of assignment tests with the GENECLASS software. Animals that could not be excluded from all reference populations at the *p* < .01‐level were assigned to their most likely population of origin. We considered the two mostly likely populations of origin if the assignment score for the first population was <85%. For further details, see Section 2. (a) Non‐outlying Belgian deer that could be excluded from the three Belgian populations using a leave‐one‐out approach; (b) non‐outlying Belgian deer that were assigned with confidence to a European reference population; (c) non‐outlying Belgian deer that had a recent non‐native ancestor; (d) Belgian outliers that could be excluded from the three Belgian reference populations; (e) non‐excluded Belgian outliers that could be assigned with confidence to a European reference population. Max. Belgium = the maximum exclusion probability observed in any of the three Belgian reference clusters. Max. Exclusion Europe = the maximum exclusion probability observed in any of the 19 European reference clusters. Animals in bold may have migrated naturally into the study area. The geographic location of the reference clusters is given in Fig. 2

ID	FCA‐outlier?	Max. Belgium	Max. exclusion Europe	Results of assignment tests			
				Most likely source	Score (%)	2nd most likely source	Score (%)
(a)							
1518	No	<0.0001	0.0037	—	—	—	—
1558	No	0.0002	0.0001	—	—	—	—
137	No	0.0021	0.0002	—	—	—	—
1258	No	0.0037	0.0001	—	—	—	—
1282	No	0.0038	<0.0001	—	—	—	—
1706	No	0.0057	<0.0001	—	—	—	—
877	No	0.0066	0.0233	NE Ger E Pol	99.13		
348	No	0.0089	0.0014	—	—	—	—
(b)							
**972**	**No**	**0.0175**	**0.0231**	**E France**	**100**	—	—
**1017**	**No**	**0.0219**	**0.1806**	**E France**	**98.53**	—	—
1386	No	0.0522	0.8498	NE Ger E Pol	77.09	NW France	13.93
1028	No	0.2640	0.8524	NE Ger E Pol	100	—	—
699	No	0.5077	0.6272	NW Croat S Slo	99.94	—	—
(c)							
1652	No	0.0203	0.0283	Belgium 1	51.79	E Germany	38.24
**224**	**No**	**0.0304**	**0.0836**	**Belgium 1**	**58.07**	**E France**	**37.72**
**1159**	**No**	**0.0390**	**0.0470**	**Belgium 1**	**71.23**	**E France**	**25.94**
347	No	0.0760	0.4693	Belgium 1	68.18	NE Ger E Pol	30.01
**221**	**No**	**0.0825**	**0.0435**	**Belgium 1**	**75.60**	**E France**	**16.49**
1039	No	0.1252	0.2883	Belgium 1	77.87	Liecht & N Italy	18.86
47	No	0.1884	0.3488	Belgium 1	61.46	Liecht & N Italy	35.01
1097	No	0.1992	0.7284	Belgium 1	69.39	NE Ger E Pol	24.56
1771	No	0.4116	0.9330	Belgium 1	54.87	NE Ger E Pol	24.70
339	No	0.6925	0.7328	Belgium 1	83.66	Scotland	15.43
(d)							
1024	Yes	<0.0001	0.0049	—	—	—	—
1510	Yes	0.0001	0.0096	—	—	—	—
458	Yes	0.0002	0.0003	—	—	—	—
1517	Yes	0.0006	0.0011	—	—	—	—
1613	Yes	0.0011	0.0018	—	—	—	—
301	Yes	0.0050	0.0001	—	—	—	—
1557	Yes	0.0093	0.0097	—	—	—	—
1546	Yes	<0.0001	0.0104	NE Ger E Pol	99.36	—	—
638	Yes	<0.0001	0.0191	NE Ger E Pol	94.80	—	—
1353	Yes	<0.0001	0.0450	Deer Farm	99.98	—	—
1430	Yes	<0.0001	0.0459	NE Ger E Pol	99.99	—	—
1431	Yes	<0.0001	0.0566	Deer Farm	94.38	—	—
1438	Yes	<0.0001	0.0595	NE Ger E Pol	97.85	—	—
1384	Yes	<0.0001	0.1209	NE Ger E Pol	84.65	Scotland	15.19
1437	Yes	<0.0001	0.1296	Scotland	99.92	—	—
1027	Yes	<0.0001	0.2515	Deer Farm	83.50	Liecht & N Italy	15.43
1307	Yes	<0.0001	0.4533	Deer Farm	80.27	NE Ger E Pol	19.56
1105	Yes	<0.0001	0.4754	Deer Farm	85.24	NE Ger E Pol	14.37
1111	Yes	<0.0001	0.4983	NE Ger E Pol	99.42	—	—
1436	Yes	<0.0001	0.5163	Deer Farm	99.9	—	—
1026	Yes	<0.0001	0.6201	NE Ger E Pol	84.73	Deer Farm	15.18
1112	Yes	<0.0001	0.6875	Scotland	99.84	—	—
1428	Yes	<0.0001	0.8778	NE Ger E Pol	99.70	—	—
1031	Yes	0.0001	0.0726	E Germany	91.36	—	—
1337	Yes	0.0001	0.1945	NE Ger E Pol	99.99	—	—
1332	Yes	0.0001	0.2650	E Germany	93.60	—	—
1023	Yes	0.0001	0.6109	Scotland	71.25	NE Ger E Pol	28.07
1387	Yes	0.0003	0.7576	Deer Farm	79.22	NE Ger E Pol	20.63
8	Yes	0.0004	0.0276	NE Ger E Pol	97.97	—	—
1030	Yes	0.0006	0.0416	Liecht & N Italy	62.35	—	—
1079	Yes	0.0009	0.4282	NE Ger E Pol	100.00	—	—
1648	Yes	0.0014	0.0178	NE Ger E Pol	94.24	—	—
1653	Yes	0.0015	0.6697	Liecht & N Italy	49.61	NE Ger E Pol	44.99
1193	Yes	0.0016	0.1090	NE Ger E Pol	97.33	—	—
1435	Yes	0.0026	0.2086	Liecht & N Italy	68.95	Scotland	28.44
1389	Yes	0.0029	0.8207	Deer Farm	78.28	NE Ger E Pol	18.85
886	Yes	0.004	0.0551	Deer Farm	100.00	—	—
359	Yes	0.0042	0.0272	NE Ger E Pol	79.88	Cent France	20.03
1172	Yes	0.0096	0.0118	NE Ger E Pol	52.65	NW Croat S Slo	45.99
1718	Yes	0.0097	0.1169	NE Ger E Pol	99.91	—	—
(e)							
1110	Yes	0.0120	0.4078	NE Ger E Pol	53.30	Carpathians	41.38
1137	Yes	0.0107	0.5543	NE Ger E Pol	87.56	Belgium 3	12.30
1432	Yes	0.0404	0.8391	Deer Farm	91.72	NE Ger E Pol	6.45

**Figure 4:**
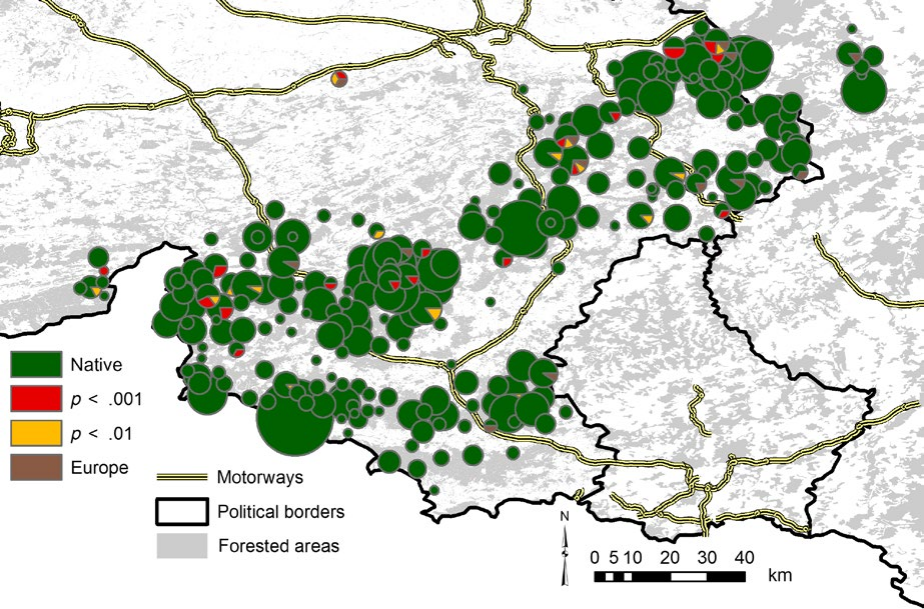
Geographic location of non‐native deer (*N* = 66). Red: animals excluded from all three Walloon STRUCTURE clusters (see Fig. 1) at the *p* < .001 level. Orange: animals excluded at the *p* < .01 level. Brown: animals not excluded from Belgium, but assigned with high confidence to a European reference population. The size of the pie charts indicates the number of samples collected from a locality. The entirely non‐autochthonous population is in the upper central part of the map directly south of a motorway

**Figure 5:**
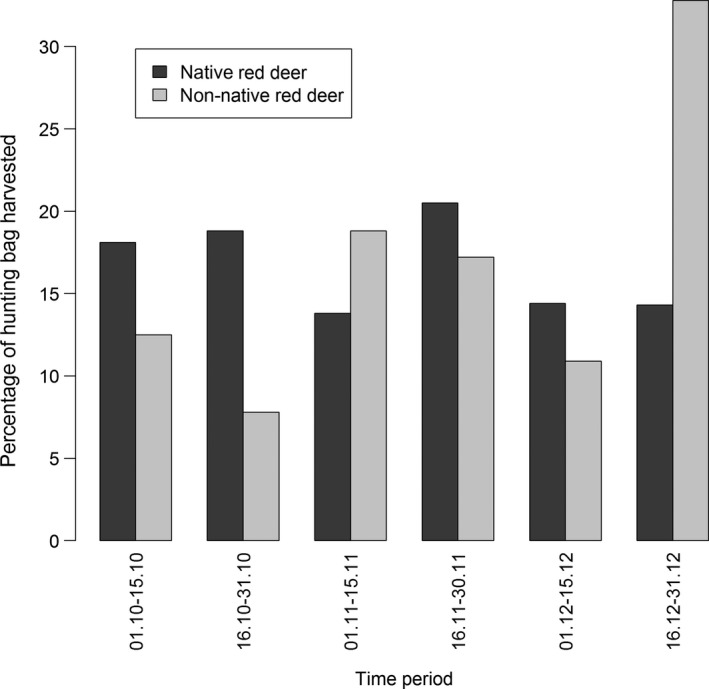
Distribution of harvest dates for studied red deer. Data included animals harvested between 2003 and 2009. Graph based on 1,638 native and 64 non‐autochthonous animals for which harvest dates were available

Altogether 40 of the 89 FCA‐outliers could be excluded from all three Walloon clusters at the *p *<* *.01‐level (28 animals even with *p *<* *.001). Of these, seven animals could also be excluded from all European clusters. The remaining samples were mostly assigned to the NE Germany/Eastern Poland cluster, the deer farm, and Scotland (Table 1). Two animals that could not be excluded from Belgium, were, however, assigned to the deer farm and NE Germany/Eastern Poland, respectively, with one further animal having a mixed European ancestry (Table 1). The 46 remaining FCA‐outliers were assigned to one (or more) Belgian cluster(s) and therefore likely to be autochthonous. Altogether, 66 (23 + 40 + 3), or 3.7%, of the 1,780 tested red deer (61 or 3.4% when neglecting the possible natural immigrants) were therefore identified as being non‐autochthonous or as having recent non‐autochthonous ancestors. A small population of deer in the northeast of the study area some distance away from the nearest regular population appears to be entirely non‐autochthonous (Fig. 4). One non‐native individual was observed in the German part of our study region. Generally, the non‐native animals occurred across most of the study area (few non‐autochthonous deer were observed in southwestern Wallonia) rather than being concentrated in a few localities (Fig. 4). While non‐autochthonous deer were harvested during the whole hunting season (21st Sep. to 31st Dec.), there was a clear increase in non‐natives toward closed season (Fig. 5). This increase was not observed with native deer.

## 4 DISCUSSION

Translocations of game animals are often carried out, sometimes illegally (see Frantz et al., 2006), for sporting purposes and to increase trophy quality (in the case of red deer: body mass, size, and number of points in antlers), but apart from blurring natural genetic patterns the potential transmission of diseases is also an imminent danger (Ferroglio et al., 2011). Until recently, the unequivocal identification of non‐autochthonous red deer and their potential origin within a geographically limited region was impeded by the lack of a continent‐wide nuclear analysis of the species and difficulties in inter‐laboratory comparisons of microsatellite alleles. We genotyped 1,780 red deer from Belgium and the Eifel at 13 microsatellite loci and compared these profiles with a European red deer dataset. The combined data of almost 2,500 red deer microsatellite profiles enabled us for the first time to quantitatively assess the proportion of non‐autochthonous red deer within a geographically limited region based on nuclear genetic markers. Given the absence of a European reference dataset, Frantz et al. (2006) were not able to confidently identify anthropogenic introductions. The present study is therefore, to our knowledge, the first to address this topic in such detail and with such an extensive dataset.

Our dataset confirmed the conclusions by Frantz et al. (2012) that the E411 motorway in the west of our study area represented a gene flow barrier to red deer. The motorways in the eastern part of our Belgium study area did not appear to have the same effect (Fig. 2). A more detailed landscape genetic study will be necessary to further untangle the effect of geographic distance and landscape features on the population genetic structure of Belgian red deer. The information provided in this study will help to avoid analytical problems related to the inclusion of non‐autochthonous animals.

The inferred frequency of non‐autochthonous individuals was between three and four percent. While this value seems rather low, it corresponds roughly to one in 25–30 red deer, which in total amounts to a large number of animals—approx. 400 based on a census size of roughly 11,000 head in Wallonia. Some of these non‐native animals may have immigrated naturally from adjoining populations (e.g., Eastern France), but our results suggest that their numbers were small compared to human‐mediated introductions (Table 1). However, there has been at least one documented case of a recreational hunter using carcasses from farmed deer to augment his hunting bag when not fulfilling his mandatory hunting quota. The observed peak in the harvest of non‐natives toward the end of the hunting season suggests that this was not an isolated case. Even allowing for these farm animals, our results are nevertheless indicative of a significant anthropogenic impact on this central European population.

We may have underestimated translocation activities, since our marker system is unlikely to have enough resolution to identify all non‐natives or descendants of non‐native animals. The markers per se have proved suitable for the study of both small‐scale and large‐scale genetic structuring and for the identification of immigration/introduction events (Dellicour et al., 2011; Frantz et al., 2008, 2012; Zachos et al., 2016). Still, analyzing a higher number of loci may have pushed a (small) number of individuals below an exclusion threshold. More generally, a microsatellite dataset will only yield information on events involving a few recent generations, while incidents further back in time are unlikely to be detected (e.g., Frantz et al., 2013). It is known that red deer have been translocated for many centuries (Apollonio et al., 2014; Niethammer, 1963), and in many cases these interferences with the natural distribution pattern and its demographic, genetic, and evolutionary consequences cannot be uncovered anymore by means of molecular (or any other) approaches.

It is not clear to what extent our thresholds (FCA‐outliers, exclusion, and assignment tests) for identifying non‐autochthonous animals were appropriate to produce an accurate estimate of the number of non‐native deer. We failed to detect some non‐autochthonous animals by excluding the 5%‐FCA‐outliers from the dataset. However, our results suggest that, in principle, the approach allows the identification of introduced animals in the absence of a priori suspects (which was the case in Frantz et al., 2006). Removing a larger percentage of all individuals might be necessary to ensure that no non‐native animals remain non‐included. Only considering animals with an exclusion probability of *p *<* *.001 as being non‐native would have reduced our estimate of the number of non‐natives, but would in all likelihood have been too conservative (e.g., Frantz et al., 2006). Considering animals that were assigned with a score of >0.85 to a Belgian population appeared a good compromise between accuracy and efficiency in the identification of admixed individuals, but may have overestimated the number of admixed individuals while not identifying all admixed individuals (Vähä & Primmer, 2006). However, while an individual threshold might be subject to debate, most animals fulfilled more than one criterion that classified them as non‐native.

Our analyses revealed two populations as the most likely source for most of the non‐autochthonous red deer in Belgium: NE Germany/Eastern Poland and the deer farm. The genetic similarity between Germany and Eastern (rather than Western) Poland can be explained by a number of documented translocations from Germany into the northeast of Poland at the end of the nineteenth and early twentieth centuries (see German and Polish references in Niedziałkowska et al., 2011). The deer farm carried the strongest genetic signals from Scottish deer on the one hand and deer from NE Germany/E Poland on the other (see Fig. 2). As a result, based on the available data, direct introductions from deer farms—and therefore indirectly from the British Isles, Germany, and Poland—seem mostly likely. While we do not have written sources on introductions from these regions into Belgian populations, this is at least in line with the fact that both the British Isles and Germany/Poland have frequently been used as source populations for translocations throughout Europe (and beyond; see e.g., Niethammer, 1963).

Clandestine translocations of game species by private individuals do, by their very nature, not follow quarantine guidelines. While the risk of disease introduction depends on the source of the introduced animals, farm‐raised ungulates are particularly prone to carry infectious diseases (Ayanegui‐Alcerreca et al., 2007; Miller & Thorne, 1993; Woodford & Rossiter, 1993). There are examples of the introduction of destructive pathogens into native populations as a result of legal translocation projects (Cunningham, 1996; Woodford & Rossiter, 1993). While in our case it might not be possible to differentiate between intentional introduction and unintentional escape of red deer from Walloon deer farms, both are likely to increase the risk of disease introduction.

If our results were representative for other regions—at least with respect to the order of magnitude—they would indicate a substantial anthropogenic impact on populations of one of the most widespread large European mammals. While results by Frantz et al. (2006) suggest that the results in the present study might be high compared to other regions, a few south‐eastern populations in our European reference dataset appeared to contain non‐autochthonous animals (see Fig. 2). European‐wide phylogeographic studies of red deer based on mtDNA have shown that the (inferred) postglacial genetic pattern of the three lineages (A, B, and C) has generally not been blurred by among‐lineage translocations (Ludt et al., 2004; Niedziałkowska et al., 2011; Skog et al., 2009). The bi‐parentally inherited nuclear microsatellites were expected to show less pronounced geographic structuring across Europe because of their higher mutation rates that result in a faster erosion of postglacial demographic signals than with mtDNA. Surprisingly, Zachos et al. (2016) found a clear signal of three genetic clusters at the highest hierarchical level in their multi‐locus dataset that was geographically congruent with the one known from mitochondrial DNA phylogeography.

Our present study with high enough resolution power (i.e., a sufficient number of loci and large sample sizes) suggests that, at smaller scales than those usually addressed in phylogeographic analyses, natural structures have been blurred to a certain extent by the introduction of non‐native individuals that subsequently reproduced successfully. This is likely to hold true not just for the Belgian red deer, but also for other European populations, including those of other ungulate species.

## CONFLICT OF INTEREST

None declared.

## AUTHOR CONTRIBUTIONS

ACF, MCF, and SB designed the study. SB and MC collected samples. MCE and MCF generated the raw data in the laboratory. ACF, FEZ, and MCF analyzed the data. All authors contributed to the writing of the manuscript.

## DATA ACCESSIBILITY

Sample locations and microsatellite genotypes: DRYAD entry https://doi.org/10.5061/dryad.145g7.
