# Supplementary material for: Using genetic tools to estimate the prevalence of non‐native red deer (Cervus elaphus) in a Western European population
Source: Ecol Evol. 2017 Aug 18;7(19):7650–60. doi: 10.1002/ece3.3282 (PMC5632609; doi:10.1002/ece3.3282)
Supplement: Supplementary file 1 [file ECE3-7-7650-s001.docx]

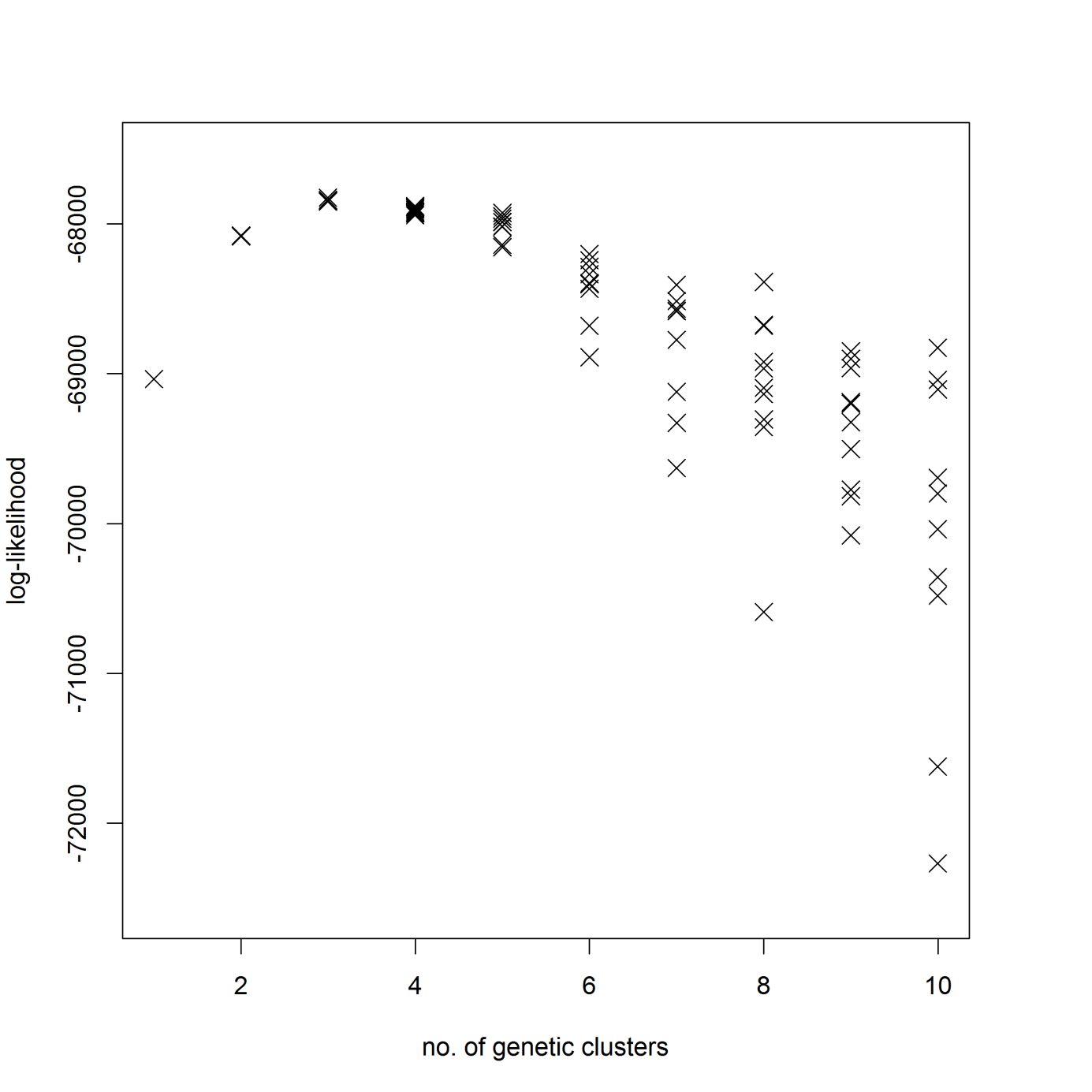


Fig. S1. Inference of genetic clusters using the STRUCTURE algorithm. Plot of the number of genetic clusters tested against their estimated log-likelihood. STRUCTURE was run using the admixture and correlated allele frequencies models.

Table S1: Significance values of GENEPOP exact test for Hardy-Weinberg deviations in the clusters derived using both the STRUCTURE and the BAPS algorithm. Values in bold are significant before correction for multiple tests, the values marked with an asterisk are significant after this correction.

| Locus |  | Structure | | |  | spatial BAPS | | |
| --- | --- | --- | --- | --- | --- | --- | --- | --- |
|  |  | Cluster 1 | Cluster 2 | Cluster 3 |  | Cluster 1 | Cluster 2 | Cluster 3 |
| BM1818 |  | 0.5575 | 0.6241 | 0.1706 |  | 0.2248 | 0.6156 | 0.2815 |
| Cer14 |  | 0.0981 | **0.0019*** | 0.4493 |  | **0.005*** | **0.0012*** | 0.2684 |
| CSPS115 |  | 0.2135 | 0.3902 | **0.0001*** |  | 0.0776 | 0.0831 | 0.0628 |
| CSSM14 |  | 0.5301 | 1.0000 | 0.2505 |  | 0.5663 | 1.0000 | **0.0440** |
| CSSM16 |  | 0.4903 | 0.2818 | 0.1993 |  | 0.3536 | 0.4982 | 0.1509 |
| CSSM19 |  | **0.0086** | 0.2859 | 0.3382 |  | **0.0047*** | 0.4290 | 0.0624 |
| CSSM22 |  | 0.2657 | 0.8897 | 0.1448 |  | 0.3597 | 0.8290 | 0.4600 |
| CSSM66 |  | **<0.0001*** | **<0.0001*** | **0.0247** |  | **<0.0001*** | **0.0025*** | **0.0194** |
| ETH225 |  | 0.3527 | 0.5607 | 0.4669 |  | 0.4996 | 0.7459 | 0.2858 |
| Haut14 |  | **0.0488** | 0.2475 | 0.589 |  | 0.0749 | **0.0360** | 0.3477 |
| ILSTS06 |  | 0.3184 | 0.3969 | 0.2089 |  | 0.0661 | 0.0695 | **0.0317** |
| INRA35 |  | 0.1374 | 0.7517 | 0.9076 |  | 0.3111 | 0.6851 | 0.7034 |
| MM12 |  | 0.3656 | 0.5605 | **0.0445** |  | 0.0625 | 0.9235 | 0.2535 |
